# Effect of Bismuth Oxide on the Microstructure and Electrical Conductivity of Yttria Stabilized Zirconia

**DOI:** 10.3390/s16030369

**Published:** 2016-03-14

**Authors:** Liwei Liu, Zheng Zhou, He Tian, Jixue Li

**Affiliations:** 1 State Key Laboratory of Silicon Materials, Department of Materials Science and Engineering, Zhejiang University, Hangzhou 310027, China; llwdahai@zju.edu.cn (L.L.); zhouzhengaa@zju.edu.cn (Z.Z.); 2 Center of Electron Microscopy, Zhejiang University, Hangzhou 310027, China

**Keywords:** solid electrolyte, ionic conductivity, YSZ, bismuth oxide

## Abstract

Bismuth oxide (Bi_2_O_3_)-doped yttria-stabilized zirconia (YSZ) were prepared via the solid state reaction method. X-ray diffraction and electron diffraction spectroscopy results indicate that doping with 2 mol% Bi_2_O_3_ and adding 10 mol% yttria result in a stable zirconia cubic phase. Adding Bi_2_O_3_ as a dopant increases the density of zirconia to above 96%, while reducing its normal sintering temperature by approximately 250 °C. Moreover, electrical impedance analyses show that adding Bi_2_O_3_ enhances the conductivity of zirconia, improving its capability as a solid electrolyte for intermediate or even lower temperatures.

## 1. Introduction

Because of its good high-temperature electrical conductivity, thermal stability, and chemical stability, 8 mol% yttria-stabilized zirconia (8YSZ) ceramic material has been used as an oxygen ion conductor in oxygen sensors [1,2,3,4] and solid oxide fuel cells [5,6,7]. However, the electrical conductivity of this type of material at intermediate temperatures around 600 °C needs to be improved in order to enhance the stability, sensitivity of electrochemical devices and lower the energy consumption [8,9,10,11]. In addition, during the production of 8YSZ, the sintering temperature reaches as high as about 1550 °C, causing manufacturing difficulties. Therefore, it is desirable to reduce the sintering temperature of 8YSZ as well as increase its conductivity [1,12,13,14].

Using Bi_2_O_3_ as a sintering aid during sintering can effectively reduce the sintering temperature of zirconia, as well as increase its density. Hirano *et al*. [15] reported that doping with 1 mol% Bi_2_O_3_ decreases the sintering temperature of scandia-stabilized zirconia ceramics by 300 °C, while allowing its electrical conductivity at 1000 °C and 800 °C to reach 0.33 and 0.12 S/cm, respectively. Yeh *et al*. [12] studied the effect of bismuth doping on YSZ sintering and they discovered that small amounts of Bi_2_O_3_ were able to effectively reduce sintering temperature and promote ceramic densification. Besides serving as a sintering aid, Bi_2_O_3_ is also a very good oxygen ion conductor, and adding Bi^3+^ to zirconia can also produce oxygen vacancies. Bai *et al*. [16] studied the effect of Bi_2_O_3_ on the physical and electrochemical properties of scandia-stabilized zirconia, and they found that the conductivity of samples doped with 2 mol% Bi_2_O_3_ was also enhanced. Similar results were also discovered by Raghvendra and Prabhakar Singh in Bi_2_O_3_ doped calcia-stabilized zirconia [9] and Sara *et al*. [17]. Winnubst *et al*. [18] studied the effects of varying amounts of Bi_2_O_3_ on the ionic conductivity of YSZ, but in those studies it was found that the enhancing effect of Bi_2_O_3_ on the ionic conductivity of YSZ was very limited. Verkerk *et al*. [19] discovered that due to the emergence of numerous monoclinic phases, Bi_2_O_3_ has a negative influence on the electrical conductivity of YSZ, but they did not study the properties of Bi_2_O_3_ doped YSZ in a completely stable cubic phase [12]. Overall the effects which Bi_2_O_3_ can exert on the electrical conductivity of YSZ are not well understood.

In addition, some researchers [20,21] have studied the importance of cation disorder upon aliovalent doping and its effects on the enhancement of ion positional disorder in oxides with fluorite structures like YSZ. It has been previously reported that this positional disorder could result in higher ionic conductivity in solid electrolyte materials. Therefore, we hoped that the addition of Bi_2_O_3_ might also have a influence like that and be beneficial to the improment of the conductivity of YSZ.

This study investigates the influence of Bi_2_O_3_ on the polymorphic phasing and ionic conductivity of YSZ via X-ray diffraction (XRD), electron diffraction, electron microscopy, dielectric spectroscopy, *etc*. Then we report the effects of bismuth oxide doping on reducing zirconia sintering temperature, increasing the density and conductivity.

## 2. Experimental Section

In this experiment, zirconia powder (99.9%, Aladdin Industrial Corporation, Shanghai, China) was used as a reactant in the solid state reaction to prepare Bi_2_O_3_ doped YSZ. 8 mol% YSZ (8YSZ) and 10 mol% YSZ (10YSZ) were each mixed with 2 mol% [17,22,23] Bi_2_O_3_ powder (99.9%, Aladdin Industrial Corporation, Shanghai, China), adding ethanol as an abrasive, and ground in a planetary ball mill for 10 h. The ground powder was then pressed into disks with a diameter of 10 mm and a thickness of 2 mm via cold isostatic pressing. The disks were then sintered in air atmosphere for 2 h at 1100, 1200, 1300, and 1400 °C, respectively. An 8YSZ ceramic disk was sintered at 1550 °C for 4 h as a control.

The density of the ceramic disks was determined via the Archimedes method. Crystal structure was studied via X-ray diffraction (X'Pert PRO, PANalytical, Almelo, the Netherlands). Microscopic morphology was characterized with a scanning electron microscope (SEM, SU-70, Hitachi, Tokyo, Japan). Samples for transmission electron microscopy (TEM) and spectroscopy were prepared via a dual beam-focused ion beam (FIB, Quanta 3D FEG, FEI, Hillsboro, OR, USA) instrument. A G2 F20 transmission electron microscope (FEI Tecnai, Hillsboro, OR, USA) was used to perform TEM and high angle annular dark field scanning transmission electron microscopy (HADDF-STEM) analysis on Bi10YSZ samples sintered at 1300 °C. X-ray Energy Dispersive Spectroscopy (EDS) data was acquired at STEM model. For electrodes, the sample disks were first painted with a thin and homogeneous silver slurry lamella as big as the disk surface, and then sintered at 700 °C for 30 min to get bright and smooth silver electrodes. The electrical properties of the samples were measured using a VersaSTAT electrochemical workstation (Ametek, Bowen, PA, USA) at 50 °C intervals from 350 °C to 650 °C, with the frequency range of 0.1 Hz–1 MHz and a voltage of 10 mV.

## 3. Results and Discussion

### 3.1. Structural Characterization

**Figure 1:**
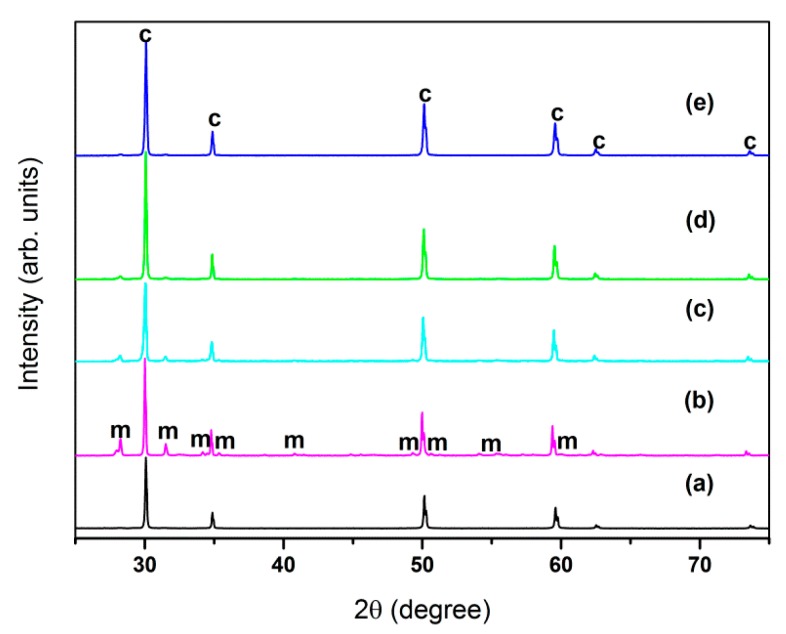
XRD pattern: (**a**) 8YSZ 1550 °C; (**b**) 2Bi-8YSZ 1100 °C; (**c**) 2Bi-8YSZ 1200 °C; (**d**) 2Bi-8YSZ 1300 °C; (**e**) 2Bi-8YSZ 1400 °C.

Figure 1 is the XRD diffraction pattern of the 2 mol% Bi_2_O_3_-doped 8YSZ (2Bi-8YSZ) samples sintered at varying temperatures and 8YSZ sintered at 1550 °C. Diffraction shows that the primary structure of the sample is cubic phase (c), with small amounts of the monoclinic phase (m). The monoclinic phase exhibits low electrical conductivity and causes material instability due to the polymorphic transitions, thereby making it an undesirable phase for zirconia as a solid electrolyte material. The purpose of the stabilization with yttria is to reduce and eliminate the monoclinic phase in zirconia. Table 1 shows the estimated weight percent of monoclinic phases in different samples for reference. The weight percent of cubic phases is calculated by the internal standard method with the values of RIR and the integrated intensity of the the strongest peak in both phases base on the XRD data. As Figure 1 and Table 1 show, the quantity of monoclinic phase is gradually reduced as the sintering temperature is increased. The 8YSZ sample was in complete cubic phase at the sintering temperature of 1550 °C, while the 8YSZ doped with 2 mol% Bi_2_O_3_ failed to produce a zirconia sample with a complete cubic phase.

**Figure 2:**
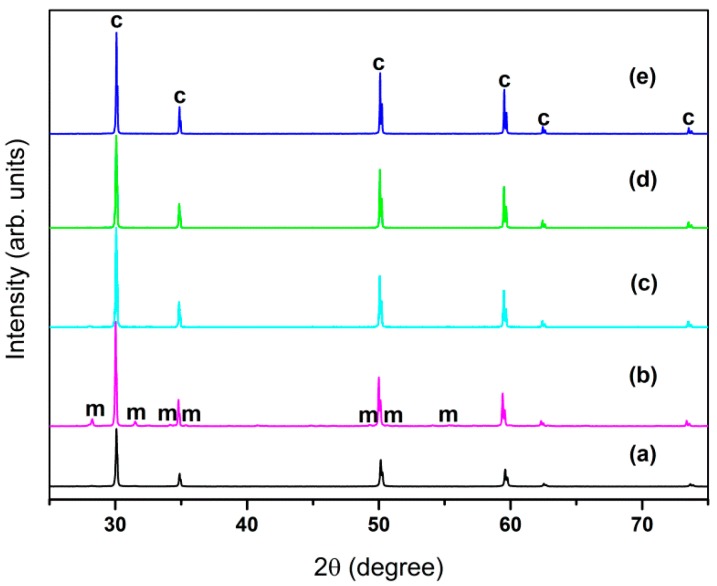
XRD pattern: (**a**) 10YSZ 1550 °C; (**b**) 2Bi-10YSZ 1100 °C; (**c**) 2Bi-10YSZ 1200 °C; (**d**) 2Bi-10YSZ 1300 °C; (**e**) 2Bi-10YSZ 1400 °C.

Figure 2 is the XRD diffraction pattern of 2Bi-10YSZ sintered at various temperatures and 10YSZ sintered at 1550 °C, and shows that once the sintering temperature exceeds 1300 °C, 2Bi-10YSZ no longer contains the monoclinic ZrO_2_; these results were also verified via selected area electron diffraction analysis.

### 3.2. Microstructure

**Figure 3:**
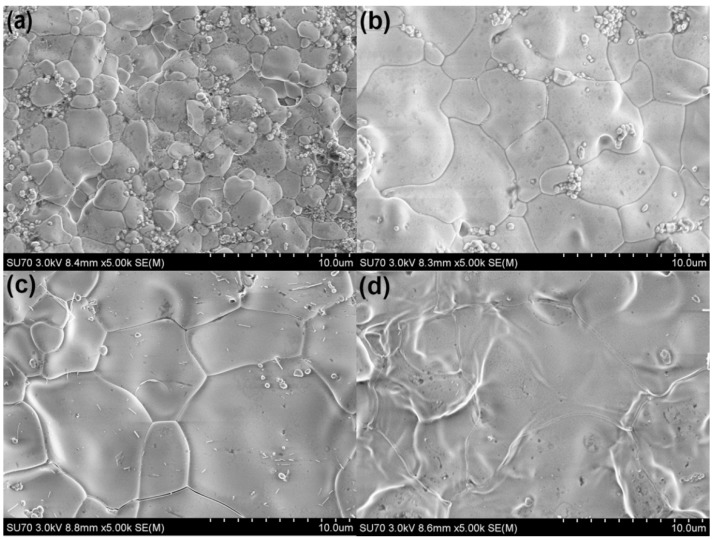
Scanning electron microscope images of 2Bi-8YSZ at various temperatures: (**a**) 1100 °C; (**b**) 1200 °C; (**c**) 1300 °C; (**d**) 1400 °C.

Figure 3 shows scanning electron microscope images of 2Bi-8YSZ sintered at various temperatures. The images indicate that the samples are composed of densely packed grains. Cavity defects decreased with the increased sintering temperatures, and the average grain size increased from about 2.5 µm to 13 µm as the sintering temperatures increased.

**Figure 4:**
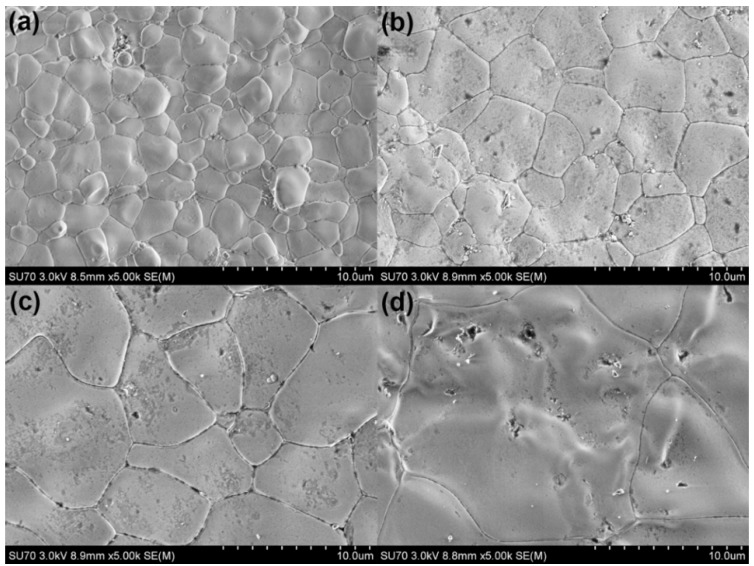
Scanning electron microscope images of 2Bi-10YSZ at various temperatures: (**a**) 1100 °C; (**b**) 1200 °C; (**c**) 1300 °C; (**d**) 1400 °C.

Figure 4 is a scanning electron micrograph of 2Bi-10YSZ sintered at various temperatures. The image indicates that the 2Bi-10YZ sample has a more uniform morphology as well as fewer small second phase grains. These results are consistent with the XRD analysis results, which all indicate that more yttria (10 mol%) is needed in the system to stabilize zirconia and obtain a uniform, fully stabilized cubic structure. This dense and void-free morphology suggest that adding low-melting Bi_2_O_3_ can significantly improve material density and promote grain growth because of introducing the liquid phase sintering effect [9,12,18]. The rounded rather than straight grain boundaries seen in the microstructure can be explained as the results of border infiltration occurring during the liquid sintering process. Table 2 shows the different grain size and density of samples sintered at different temperatures. Doping with Bi_2_O_3_ improved the sample density, and the grain size increased as the sintering temperature increased. Analysis of the XRD spectra and the SEM picture show that 2Bi-10YSZ can form stabilized zirconia with a uniform, dense, and complete cubic phase structure. This type of homogeneous, stable full cubic zirconia will likely exhibit better electrical properties and stability.

### 3.3. Transmission Electron Microscopy and EDS Analysis

**Figure 5:**
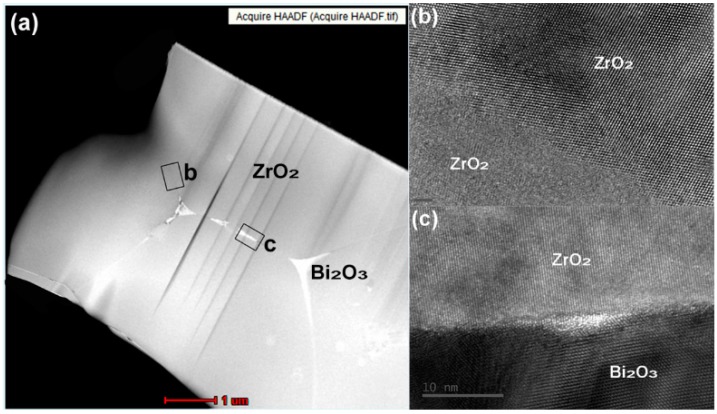
2Bi-10YSZ sample sintered at 1300 °C: (**a**) HADDF-STEM image; (**b**,**c**) high-resolution images of grain boundaries.

As Figure 5a shows, second phase precipitation appeared at triangular grain boundaries and some grain boundaries. Bi_2_O_3_ presented at the grain boundaries can act as fillers in the triangular boundary and increase the material density with the liquid sintering. The high resolution pictures in Figure 5b,c represent the b and c areas marked with rectangles in Figure 5a. Figure 5b is a high resolution picture of the grain boundary between two zirconia grains. Figure 5c is a high resolution picture of the grain boundary between a zirconia and a Bi_2_O_3_ grain. The high resolution pictures show that there are only 2–3 atomic layers at the transition interphase between the zirconia grain boundaries, while the transition interphase is slightly widened between the zirconia and Bi_2_O_3_ grains. There was no amorphous layer in both grain boundaries. The impurities present in an amorphous layer are high resistance materials, which will block the conduction and migration of oxygen vacancies. Thus, the lack of an amorphous layer at the grain boundary can also enhance conductivity. As Figure 5c shows, the zirconia and Bi_2_O_3_ grains contact and converge nicely at the grain boundary, which makes the Bi_2_O_3_ located in the boundary looks like a bridge linking the zirconia grains together.

**Figure 6:**
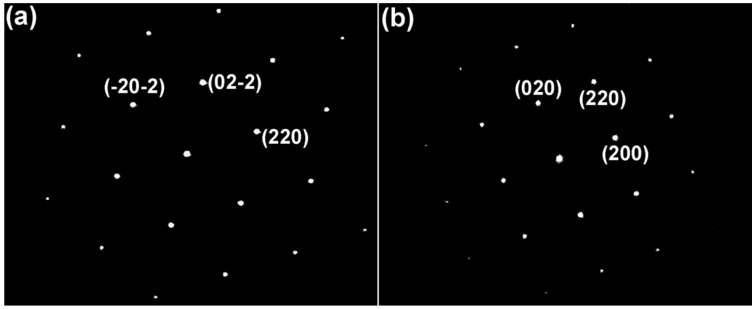
Selected area electron diffraction pattern of 2Bi-10YSZ sampled sintered at 1300 °C: (**a**) FCC zone axis [011]; (**b**) FCC zone axis [001].

**Figure 7:**
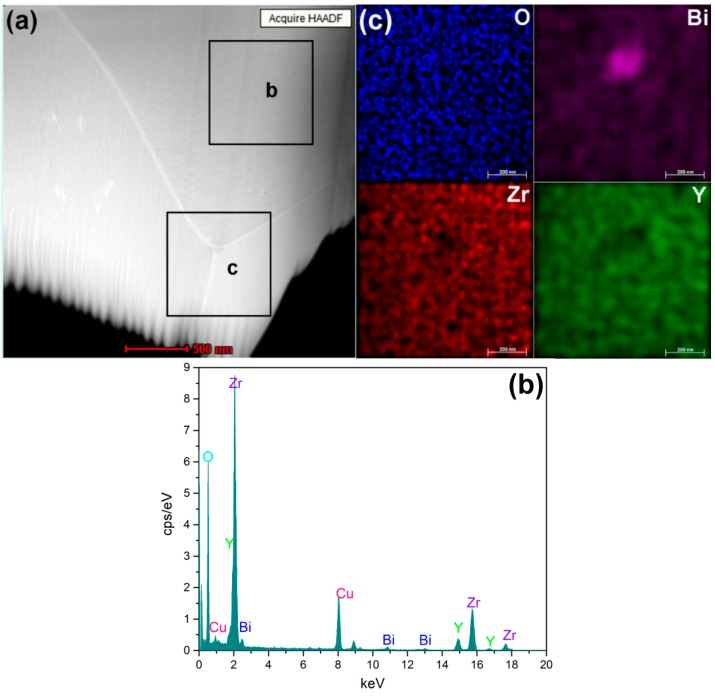
(**a**) HADDF-STEM image of 2Bi-10YSZ; (**b**,**c**) EDS energy spectrum.

Figure 6 is a selected area electron diffraction pattern of 2Bi-10YSZ sample sintered at 1300 °C. The diffraction zone axis in Figure 6a,b are the [011] and [001] cubic directions. SAED results confirm that the crystal configuration of 2Bi-10YSZ is the cubic phase structure. The spectrum shown in Figure 7b is the analysis result of the “b” area in Figure 7a, in which a bismuth peak also proves that some Bi_2_O_3_ is dissolved in the zirconia lattice, that is, Bi_2_O_3_ and yttria have a co-doping effect in the zirconia lattice. EDS spectrum analysis (Figure 7a,c) show that oxygen, yttria, and zirconium are uniformly distributed in the material without significant segregation, while bismuth segregation is mainly observed at the triangular grain boundary. Transmission electron microscopy and EDS analysis demonstrate that Bi_2_O_3_ is present in grains and grain boundaries, and enhances the density and conductivity of the material.

### 3.4. Impedance and Conductivity Measurement and Analysis

**Figure 8:**
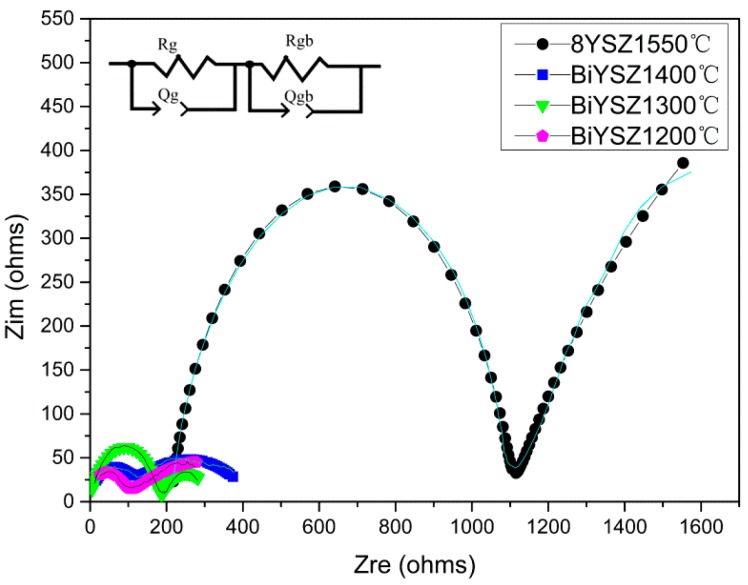
Nyquist plot of 8YSZ sintered at 1550°C and 2Bi-10YSZ sintered at various temperatures.

Figure 8 is a complex impedance plane plot, or Nyquist plot, of 8YSZ and 2Bi-10YSZ samples of different sintering temperatures tested at 500 °C. The data processing methods for all samples are the same; deduction correction was performed on the curves with the ohmic resistance contribution to allow a better comparation for the resistances of different samples. All impedance data was processed and analyzed using ZSimpWin software. The lines in Figure 8 are the experiment data and the different symbols with different colors are the fitting results. Grain or grain boundary resistance is calculated by fitting two series RQ circuit elements to the impedance spectra. The derived equivalent circuit diagram of the impedance curve is shown in the insert of Figure 8. The high frequency and low frequency semicircle of 2Bi-10YSZ sample shown in Figure 8 represent the grain resistance and grain boundary resistance [17,24,25], respectively. Due to the relaxation properties of different materials and the relatively high measurement temperature, the high-frequency semicircle of the 8YSZ sample represents its grain boundary resistance, and the grain resistance is calculated via the horizontal intercept of the high frequency impedance curve [16,25]. As for the capacitance (C), it can be calculated with the data of the constant phase element (Q). The valves of C are of the expected magnitude, *i.e*., of about 10^−10^ F and 10^−7^ F for the grains and the grain boundaries, respectively.

The figure shows that compared with the 8YSZ sample, which has almost the highest electrical conductivity among all YSZ materials [3,5,26], the 2Bi-10YSZ samples have decreased high and low frequency semicircles. In the 8YSZ sample, the bulk resistance mainly comes from the grain boundary resistance; while in 2Bi-10YSZ sample, the grain boundary resistance is close to the grain resistance. The grain boundary resistance significantly decreased in 2Bi-10YSZ, while its grain resistance also slightly decreased, indicating an improvement of the electrical properties. Similar phenomena have been reported by Raghvendra and Prabhakar Singh [9] in Bi_2_O_3_ doped calcium oxide stabilized zirconia materials; and it has also been discovered by Bai *et al*. [16] in Bi_2_O_3_-doped scandia stabilized zirconia.

**Figure 9:**
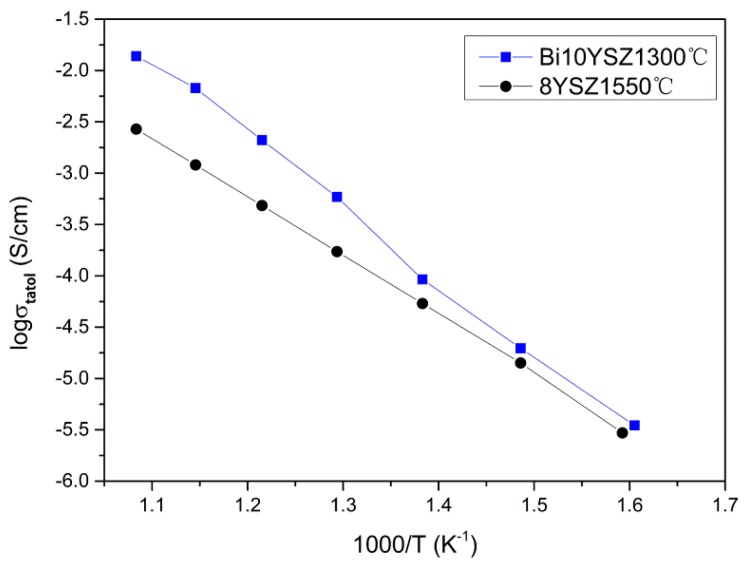
Arrhenius curve of 8YSZ sintered at 1550 °C and 2Bi-10YSZ sintered at 1300 °C.

Comparison between 2Bi-10YSZ samples of different sintering temperatures shows that the sample sintered at 1300 °C exhibits the lowest total resistance. Figure 9 is an Arrhenius curve of 2Bi-10YSZ sintered at 1300 °C and 8YSZ sintered at 1550 °C showing the ionic conductivity plotted against inverse temperature. The sample conductivity is calculated as: σ = $\frac{L}{RA}$. L and A are the thickness and circular area of the test wafer sample, respectively, and R is the resistance of grain or grain boundaries. In 2Bi-10YSZ samples, the sample sintered at 1300 °C exhibits the highest conductivity of 0.013 S/cm at 650 °C, and the conductivity of 8YSZ sintered at 1550 °C is about 0.003 S/cm at 650 °C, demonstrating that adding Bi_2_O_3_ improves the conductivity to about four times the original conductivity.

**Figure 10:**
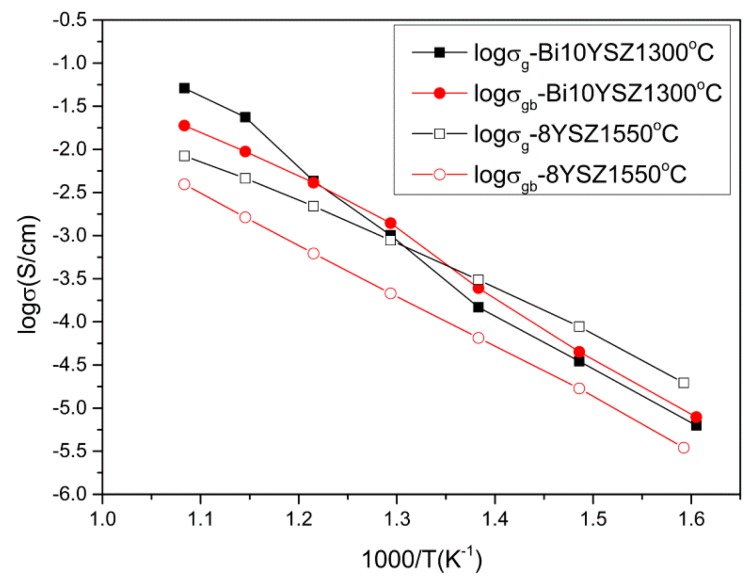
Grain and grain boundary Arrhenius curve of 8YSZ sintered at 1550 °C and 2Bi-10YSZ sintered at 1300 °C.

In Figure 10, the grain and grain boundary Arrhenius curves of 8YSZ of 1550 °C and 2Bi-10YSZ of 1300 °C show that the improvement of sample conductivity of 2Bi-10YSZ mainly occurs in the grain boundaries. When the test temperature is higher than 500 °C, the grain conductivity of Bi10YSZ is also higher than that of 8YSZ. One of the reasons for the conductivity improvement is the promotion of grain growth by Bi_2_O_3_, affording a high density material. Another important reason for the boundary improvement is attributed to the aggregation and segregation of some Bi_2_O_3_. Bismuth oxide-based electrolyte is a widely used solid electrolyte material, which has a higher ionic conductivity than zirconia-based materials. Because these materials contain more oxygen vacancies on one side. On the other side, Bi^3+^ has a high polarization capability due to the influence of 6s^2^ lone pair electrons and a strong ability to adapt and integrate to a relatively disordered surroundings [27,28,29]. Thus, Bi_2_O_3_ segregated at the grain boundaries can remove impurities, such as aluminum oxide, silicon oxide, *etc*. [30,31], so that the amorphous phase with high resistance is absent at the grain boundaries. What’s more, the Bi_2_O_3_ segregated at grain boundaries bridges the grains for oxygen transportation and increases the sample conductivity. And for the part of grain conductivity, the Bi_2_O_3_ infiltrated into the zirconia grains can also generate oxygen vacancies due to the charge compensation mechanism, of which the defect reaction can be represented by the following equation: *Bi_2_O_3_* $\overset{ZrO_{2}}{\to }2Bi_{Zr}^{\prime }$ + $3O_{O}^{X}$ + $\ddot{V}$*_O_*.

However, at low temperatures, these oxygen vacancies cannot migrate freely, and most of which will bind and interact with cations. Thus, at lower temperatures, the 2Bi-10YSZ grain conductivity has no big difference with that of 8YSZ. When the temperature becomes higher, more vacancies get free from association to migrate. The conductivity of the 2Bi-10YSZ grain increases notably in the latter range and the grain conductivity of 2Bi-10YSZ also become higher than that of 8YSZ. Table 3 shows the activation energies for grain and grain boundary of 8YSZ and Bi10YSZ. They are sintered at 1550 °C and 1300 °C separately. All the activation energies are similar to the published data [7,32]. It indicates that when Bi_2_O_3_ is added in YSZ, the activation energies increase. The increace may be ascribed to the added oxygen vacancies with more correlation effects and the lattice distortion in both the grain and grain boundary caused by the solution of Bi_2_O_3_ in the grain and the segregation of Bi_2_O_3_ in the grain boundary, respectively. Thus, there are more vacancies and defect positions in the Bi10YSZ, which is benificial to the ions transport and then the conductivity of the sample becomes more sensitive to the temperature.

## 4. Conclusions

Doping with Bi_2_O_3_ enhances the grain growth of 10YSZ, reduces its sintering temperature and improves its ionic conductivity. 10 mol% YSZ doped with 2 mol% Bi_2_O_3_ exists in a completely stable cubic phase after sintering at 1300 °C. Due to the filling effect of Bi_2_O_3_ at grain boundaries, 2Bi-10YSZ has a higher conductivity of 0.013 S/cm at 650 °C, which is about four times higher than that of 8YSZ.

## Figures and Tables

**Table 1:** The weight percent of monoclinic phases of different samples.

Sample	Sintering Temperature (°C)	Weight Percent of Monoclinic Phase (wt%)
8YSZ	1550	0
2Bi-8YSZ1	1100	33
2Bi-8YSZ2	1200	19
2Bi-8YSZ3	1300	17
2Bi-8YSZ4	1400	6
2Bi-10YSZ1	1100	20
2Bi-10YSZ2	1200	4
2Bi-10YSZ3	1300	0
2Bi-10YSZ4	1400	0

**Table 2:** Grain size and densiy of samples.

Sample	Sintering Temperature (°C)	Grain Size (µm)	Relative Density (%)
8YSZ	1550	6	96.0
2Bi-8YSZ1	1100	2.5	97.5
2Bi-8YSZ2	1200	5	97.8
2Bi-8YSZ3	1300	10	96.7
2Bi-8YSZ4	1400	13	98
2Bi-10YSZ1	1100	3	97.7
2Bi-10YSZ2	1200	5	98.0
2Bi-10YSZ3	1300	10	96.5
2Bi-10YSZ4	1400	14	98.2

**Table 3:** Activation energies of grain and grain boundary of 8YSZ and Bi10YSZ.

Sample	Activation Energy of Grain (eV)	Activation Energy of Grain Boundary (eV)
8YSZ-1550 °C	1.03	1.17
Bi10YSZ-1300 °C	1.35	1.26
